# Lagged and instantaneous dynamical influences related to brain structural connectivity

**DOI:** 10.3389/fpsyg.2015.01024

**Published:** 2015-07-21

**Authors:** Carmen Alonso-Montes, Ibai Diez, Lakhdar Remaki, Iñaki Escudero, Beatriz Mateos, Yves Rosseel, Daniele Marinazzo, Sebastiano Stramaglia, Jesus M. Cortes

**Affiliations:** ^1^Basque Center for Applied MathematicsBilbao, Spain; ^2^Biocruces Health Research Institute, Cruces University HospitalBarakaldo, Spain; ^3^Radiology Service, Cruces University HospitalBarakaldo, Spain; ^4^Department of Data Analysis, Faculty of Psychological and Pedagogical Sciences, Ghent UniversityGhent, Belgium; ^5^Dipartimento di Fisica, Universitá degli Studi di Bari and INFNBari, Italy; ^6^Ikerbasque, The Basque Foundation for ScienceBilbao, Spain; ^7^Department of Cell Biology and Histology, University of the Basque CountryLeioa, Spain

**Keywords:** structural equation modeling, functional connectivity, structural connectivity, resting state, functional magnetic resonance imaging, tensor diffusion imaging

## Abstract

Contemporary neuroimaging methods can shed light on the basis of human neural and cognitive specializations, with important implications for neuroscience and medicine. Indeed, different MRI acquisitions provide different brain networks at the macroscale; whilst diffusion-weighted MRI (dMRI) provides a structural connectivity (SC) coincident with the bundles of parallel fibers between brain areas, functional MRI (fMRI) accounts for the variations in the blood-oxygenation-level-dependent T2^*^ signal, providing functional connectivity (FC). Understanding the precise relation between FC and SC, that is, between brain dynamics and structure, is still a challenge for neuroscience. To investigate this problem, we acquired data at rest and built the corresponding SC (with matrix elements corresponding to the fiber number between brain areas) to be compared with FC connectivity matrices obtained by three different methods: directed dependencies by an exploratory version of structural equation modeling (eSEM), linear correlations (C) and partial correlations (PC). We also considered the possibility of using lagged correlations in time series; in particular, we compared a lagged version of eSEM and Granger causality (GC). Our results were two-fold: firstly, eSEM performance in correlating with SC was comparable to those obtained from C and PC, but eSEM (not C, nor PC) provides information about directionality of the functional interactions. Second, interactions on a time scale much smaller than the sampling time, captured by instantaneous connectivity methods, are much more related to SC than slow directed influences captured by the lagged analysis. Indeed the performance in correlating with SC was much worse for GC and for the lagged version of eSEM. We expect these results to supply further insights to the interplay between SC and functional patterns, an important issue in the study of brain physiology and function.

## 1. Introduction

Three different main classes of brain networks are currently investigated (Friston, 1994; Sporns et al., 2004, 2005; Bonifazi et al., 2009; Friston, 2011): networks defined by theirstructural connectivity (SC) refer to anatomical connections between brain regions; networks defined by their functional connectivity (FC) account for statistical similarities in the dynamics between distinct neuronal populations; and effective connectivity (EC) networks identify interactions or information flow between regions.

Current magnetic resonance imaging (MRI) techniques have allowed SC, FC, and EC brain networks to be measured at the macroscale. Thus, SC networks have been obtained from diffusion tensor images (DTI) and high-resolution tractography (Craddock et al., 2013) while FC networks have been obtained from correlations between blood oxygen-level dependent (BOLD) time-series (Biswal et al., 1995).

Different methods can assess EC. One possibility is the dynamic causal modeling, addressing how the activity in one brain area is affected by the activity in another area using explicit models of neural populations (Friston et al., 2003; Penny et al., 2004). Other possibilities are data-driven approaches with no further assumptions about the hemodynamic response, nor about the biophysics of the BOLD signal from individual neuron to population level. Two popular existing data-driven methods to calculate EC are Granger causality (GC) (Granger, 1969) and transfer entropy (Schreiber, 2000).

Another well-known method to calculate EC is the structural equation modeling (SEM). Although SEM assumes an implicit model (i.e., an influence matrix) (Bollen, 1989), in the present study we focus on an exploratory version of SEM (labeled eSEM) where all variables might (a priori) interact with all the others. Notice that, eSEM is by construction exploratory whilst SEM is largely confirmatory. Both SEM and eSEM are methods to calculate EC, since both methods provide directed connectivity matrices.

In this paper, we aim to bring some light in a long lasting question: How brain structure is shaped by its function, and viceversa? Or alternatively, using the language of networks: How are the three classes of networks SC, FC and EC related to each other? It is important to emphasize that, this challenging problem has not yet a clear answer for any general brain condition and data set. Here, to address this question, we will focus here in the resting brain, i.e., when the brain is not performing any goal-oriented task.

Notice that despite the simplicity of the context where these patterns of brain activity are generated, the resting brain dynamics is complex, encompassing a superposition of multiple resting state networks (RSNs) (Raichle et al., 2001; Fox et al., 2005; Raichle and Mintun, 2006; Raichle and Snyder, 2007; Raichle, 2009); each RSN underlying a different cognitive function e.g., there are visual networks, sensory-motor networks, auditory networks, default mode networks, executive control networks, and some others (for further details see for instance (Beckmann et al., 2005) and references therein).

Pioneering work showed that SC and FC are correlated to some extent (Hagmann et al., 2008; Honey et al., 2009). After these fundamental papers, some other studies made use of the combined data sets to address different aspects of brain dynamics (Fraiman et al., 2009; Cabral et al., 2011; Deco et al., 2011; Goni et al., 2013; Haimovici et al., 2013; Kolchinsky et al., 2014; Marinazzo et al., 2014; Messé et al., 2014). In this paper, both structural and functional data have been used to demonstrate to which extent the EC obtained by eSEM and the FC obtained by C and PC are similar to SC.

Previous approaches analyzed fMRI data based on SEM (Bullmore et al., 2000; Schlösser et al., 2006; Kim et al., 2007; Gates et al., 2010, 2011), dealing with subsets of candidate regions selected on the basis of prior knowledge. However, the performance of these approaches depends strongly on the correctness and completeness of the hypothesized model of connections. In the present work, eSEM is applied in an exploratory fashion to a multivariate dataset corresponding to a specific brain system consisting in 15 different regions of interest (ROIs), fully covering (with no further assumptions about the underlying connectivity) three of the well-known RSNs (Beckmann et al., 2005): The sensory-motor network (SM), the executive-control network (ExC) and the default mode network (DMN). The application of eSEM returns an influence matrix which is not symmetric (i.e., a region A can influence B differently than how B influences A) and describes fully connected directed dependencies between ROIs.

The performance achieved by eSEM in correlating with SC (thus, measusing the similarity between eSEM and SC) is also compared with FC, obtained by two other methods: the linear correlation (C) and partial correlation (PC). Unlike C, PC is commonly used to analyze direct relationships among fMRI time series with good performance (Marrelec et al., 2006; Marrelec and Benali, 2009; Maki-Marttunen et al., 2013), since network influences beyond the specific pair are removed.

Furthermore, eSEM is also applied to lagged time series to estimate a saturated, fully connected, but recursive model. Notice that bi-directional influences here are detected as cross-lagged effects. The results from this *lagged* version of eSEM are compared with those from GC. As a result, we will show that lagged methods are less related to SC measures, which implies that the dependencies found in the data on slower time scales (in comparison to instantaneous interactions) are less related to SC.

## 2. Materials and methods

### 2.1. Same-subject structure-function acquisitions

This work was approved by the Ethics Committee at the Cruces University Hospital; all the methods were carried out in accordance to approved guidelines. A population of *n* = 12 (6 males) healthy subjects, aged between 24 and 46 (33.5 ± 8.7), provided information consents before the imaging session. For all the participants, we acquired same-subject structure-function data with a Philips Achieva 1.5T Nova scanner. The total scan time for each session was less than 30 min and high-resolution anatomical MRI was acquired using a T1-weighted 3D sequence with the following parameters: TR = 7.482 ms, TE = 3.425 ms; parallel imaging (SENSE) acceleration factor = 1.5; acquisition matrix size = 256 × 256; FOV = 26 cm; slice thickness = 1.1 mm; 170 contiguous sections. Diffusion weighted images (DWIs) were acquired using pulsed gradient-spin-echo echo-planar-imaging (PGSE-EPI) under the following parameters: 32 gradient directions, TR = 11070.28 ms, TE = 107.04 ms, 60 slices with thickness of 2 mm, no gap between slices, 128 × 128 matrix with an FOV of 23 × 23 cm. Changes in blood-oxygenation-level-dependent (BOLD) T2^*^ signals were measured using an interleaved gradient-echo EPI sequence. The subjects lay quietly for 7.28 min, during which 200 whole brain volumes were obtained under the following parameters: TR = 2200 ms, TE = 35 ms; Flip Angle 90, 24 cm field of view, 128 × 128 pixel matrix, and 3.12 × 3.19 × 4.00 mm voxel dimensions.

We have shown in Diez et al. (2015a) that the relationship between SC and FC found with the data used in this study is confirmed by the MGH-USC Human Connectome Project, of much higher quality. The results we show here open the possibility to a generalization to many other data sets.

### 2.2. Data preprocessing

#### 2.2.1. Structural data

To analyze the diffusion images (dMRI), the eddy current correction was applied to overcome artifacts produced by changes in the gradient field directions of the MR scanner and subject head movement. In particular, the eddy-correct tool from FSL was used to correct both eddy current distortions, and simple head motion, using affine registration to a reference volume. After this, DTIFIT was used to perform the fitting of the diffusion tensor for each voxel, using as an input the eddy-correct output. No extra de-noising was applied in the data and our results were not wrapped to any template. Two computations were performed to transform the atlas to each individual space: (1) the transformation between the MNI template to the subject structural image (T1), and (2) the transformation between the T1 to the diffusion image space. Combining both transformations, each atlas region is transformed to the diffusion space, allowing to count the number of fibers connecting all ROIs pairs. Using the corrected data, a local fitting of the diffusion tensor was applied to compute the diffusion tensor model at each voxel. Then, a deterministic tractography algorithm (FACT) (Mori et al., 1999) was applied using TrackVis (Wang et al., 2007), an interactive software for fiber tracking.

#### 2.2.2. Functional data

The functional MRI (fMRI) data was preprocessed with FSL (FMRIB Software Library v5.0). The first 10 volumes were discarded for correction of the magnetic saturation effect and for the remaining volumes, first the movement is corrected and then, the slice-time is also corrected for temporal alignment. All voxels were spatially smoothed with a 6 mm FWHM isotropic Gaussian kernel and after intensity normalization, a band pass filter was applied between 0.01 and 0.08 Hz (Cordes et al., 2001). Finally, linear and quadratic trends were removed. We next regressed out the motion time courses, the average CSF signal, the average white matter signal and the average global signal. Finally, fMRI data was transformed to the MNI152 template, such that a given voxel had a volume of 3 mm^*^3 mm^*^3 mm.

It is important to emphasize that to remove or not the average global signal in FC studies is currently a controversial issue (Saad et al., 2012); see also http://rfmri.org/GSRDiscussion. Here, following most of the studies addressing brain FC, we have applied the global signal removal; but the situation of not applying the global signal removal has been also explored (Figure S2).

#### 2.2.3. HRF blind deconvolution

In order to eliminate the confounding effect of HRF on temporal precedence, we individuated point processes corresponding to signal fluctuations with a given signature and extracted a voxel-specific HRF to be used for deconvolution, after following an alignment procedure. The parameters for blind deconvolution were chosen with a physiological meaning according to Wu et al. (2013): for a TR equal to 2.2 s, the threshold was fixed to 1 SD (standard deviation) and the maximum time lag was fixed to 5 TR (for further details on the complete HRF blind deconvolution method and the different parameters to be used (see Wu et al., 2013). The resulting time-series, after HRF blind deconvolution, are the ones used for the calculation of EC and FC.

### 2.3. ROIs extraction

Regions of interest (ROIs) were defined by using the masks of the resting state networks (RSNs) reported in Beckmann et al. (2005), which can be downloaded from http://www.fmrib.ox.ac.uk/analysis/royalsoc8/. Note that, we are not dealing with the independent components *per se*, but with the voxels time-series localized within the masks. Similar approaches based on the RSNs masks to define ROIs have been widely used before (Tagliazucchi et al., 2012; Haimovici et al., 2013; Carhart-Harris et al., 2014; Tagliazucchi et al., 2014; Diez et al., 2015b).

**Figure 1:**
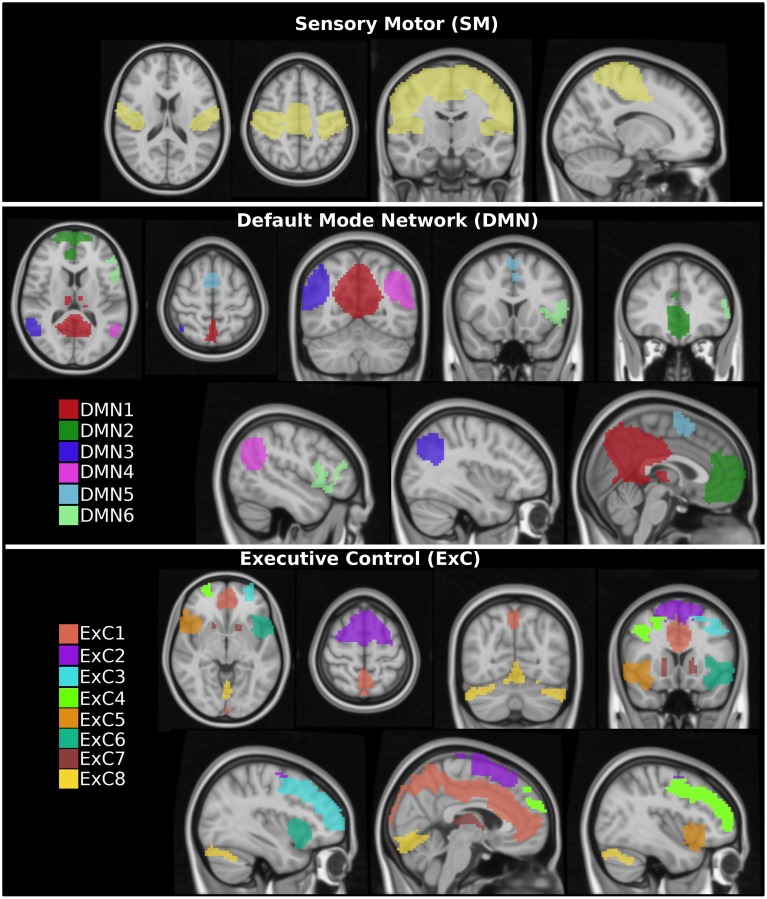
**Sketch for regions of interest (ROIs)**. Fifteen different ROIs were extracted from three different resting state networks: 1 ROI in the sensory motor (SM), 6 ROIs in the default mode network (DMN), and 8 ROIs in the executive control (ExC).

Specifically, the following three RSNs were selected: the default mode network (DMN), the executive control (ExC) network and the sensory motor (SM) network. Next, these three networks were manually subdivided in distinct spatially contiguous regions (see Figure 1). For each region, a region growing segmentation method was applied by manually selecting a seed region, thus obtaining a total of 15 different ROIs: 1 SM region, 6 DMNs and 8 ExCs regions. In particular, the “island effect” method incorporated in 3D Slicer (http://www.slicer.org) was applied, which selects all the voxels of the contiguous region given an initial seed. Visual representations of all ROIs are given in Figure 1 and their sizes in Table 1.

**Table 1:** **ROI size (mm^3^)**.

**Network**	**ROIs**	**Size (mm/^3^)**
Sensory motor (SM) network	SM	194.960
Default mode network (DMN)	DMN1	97.091
	DMN2	44.115
	DMN3	28.374
	DMN4	22.330
	DMN5	8.343
	DMN6	10.826
Executive control (ExC)	ExC1	79.956
	ExC2	43.313
	ExC3	52.225
	ExC4	48.483
	ExC5	15.769
	ExC6	15.745
	ExC7	11.723
	ExC8	32.752

### 2.4. Calculation of structural, functional and effective connectivity matrices

#### 2.4.1. Structural connectivity (SC)

Matrices were obtained per each subject by counting the number of fibers connecting two ROIs (that is, starting in one ROI and finalizing in another) for each individual pair; thus, for a number of 15 ROIs, it gave 105 different values.

#### 2.4.2. Functional connectivity (FC)

Matrices were calculated by applying to the rs-fMRI time series two methods: the linear correlation coefficient (C) and the partial correlation analysis (PC). Here, C was calculated by using the *corr* function from Matlab (MathWorks Inc., Natick, MA). Assuming C to be a non-singular matrix, the elements of the PC matrix satisfy that ${PC}_{ij}\propto {\left(C^{-1}\right)}_{ij}$, so they are proportional to the elements of the so-called precision matrix (Maki-Marttunen et al., 2013). Here, PC was computed using the *partialcorr* function from Matlab (MathWorks Inc., Natick, MA). Thus, PC is an extension of C to calculate direct interactions between pairs, as it achieves to remove for a given pair the correlation contribution from other pairs.

#### 2.4.3. Effective connectivity (EC) by the exploratory structural equation modeling (eSEM)

This refers to a statistical technique aiming to estimate Granger-causal relationships based on quantitative and qualitative causal information, by means of linear regression-based models. Unlike regression, SEM is formulated as a confirmatory model rather than a predictive model. Being interested in the description of the directed dependencies between the 15 ROIs, avoiding any prior hypothesis on the connectivity pattern, we here applied multiple regressions among all the variables. Therefore, our analysis by SEM has neither structural model nor a measurement model, and provides a fully connected estimate of the directed dependencies among all the pairs of ROIs. This exploratory analysis is referred as eSEM. This model does not use temporal correlations in the data and it is applied to non-lagged time series.

To estimate the model parameters of eSEM, a standard maximum likelihood estimation was used using the lavaan package in R (Rosseel, 2012; R Core Team, 2014). Notice that, this is justified since for saturated linear models, the maximum likelihood estimates are identical to least squares estimates.

In a second part of this study, eSEM was also applied to lagged time series to estimate a saturated, fully-connected, but recursive model. Notice that lagged eSEM is recursive but the non-lagged eSEM is not. The observed variables are the time series for the 15 ROIs augmented with lagged versions of the same time series. For eSEM1, only the time series accounting for lag = 1 were added, resulting in 30 variables in total; for eSEM2, both lag = 1 and lag = 2 time series were added, resulting in 45 observed variables in total; finally, for eSEM3, lag = 1, lag = 2, and lag = 3 time series were added, resulting in 60 observed variables in total. Three types of parameters were included in the model: (1) all autoregressive regressions within each ROI to take into account the time-dependencies; (2) all possible cross-lagged regressions between the ROIs; (3) (residual) covariances for all other pairwise relations that were not included in the set of regressions (for example, all contemporaneous connections). Importantly, contemporaneous regressions between ROIs at the same time point were not included. Moreover, to estimate the model parameters of eSEM1, eSEM2 and eSEM3, standard maximum likelihood estimation was used using the lavaan package (Rosseel, 2012).

After estimation of all model parameters, an influence matrix was computed as follows: For each pair, the evidence for this particular (directed) connection was collected. For eSEM1, this was simply the regression coefficient corresponding to the cross-lagged effect of one ROI on another (controlling for both auto-regressive effects and cross-lagged effects of other ROIs). That is, the effect of a ROI on the previous time point on a target ROI at the current time point. For eSEM2, this was a function (here, the product) of two regression coefficients: one for the effect of a ROI on the previous time point on the target ROI at the current time point (just like eSEM1), and one for the effect of a ROI measured two time points toward the target ROI at the current time point. This was done for all possible pairs, averaging all ROIs of the influence matrix except for the diagonal, which was kept at zero.

Notice that the cross-lagged evidence is only used to determine the directed influence of one ROI on another, while controlling for both auto-regressive effects and the cross-lagged effects of other ROIs. In fact, the regression coefficients computed by eSEM1, eSEM2 and eSEM3 are identical to those that would be computed when Granger causality (GC1, GC2, GC3, of order 1, 2, and 3 respectively) is employed (Granger, 1969); see also Appendix for further details. But instead of computing an *F*-statistics for each pairwise connection as GC does, here, we use the product regression coefficient(s) to average the influence matrix.

### 2.5. Statistical analysis

The values of the average matrices across subjects eSEM, C and PC were compared into two groups: values associated to structurally connected pairs (CP), meaning that two ROIs are connected with a non-zero fiber number, and those ones associated to non-connected pairs (NCP), i.e., zero fibers existed between the two ROIs. A One-Way ANOVA test was performed using the MATLAB function anova1 (MathWorks Inc., Natick, MA) between CP and NCP (statistical significance is considered to have a *p* < 0.01). Thus, small *p*-values show that the connectivity matrices calculated on the two groups CP and NCP have a different mean, i.e., they are different from each another which indicates that a given method can separate connected pairs from non-connected ones. The same analysis was also applied to eSEM1, eSEM2, eSEM3, GC1, GC2, GC3.

## 3. Results

Firstly, the three different resting networks SM, DMN, and ExC were selected. Next, the three networks were divided in a total number of 15 different ROIs (see Section 2 and Figure 1 for details).

**Figure 2:**
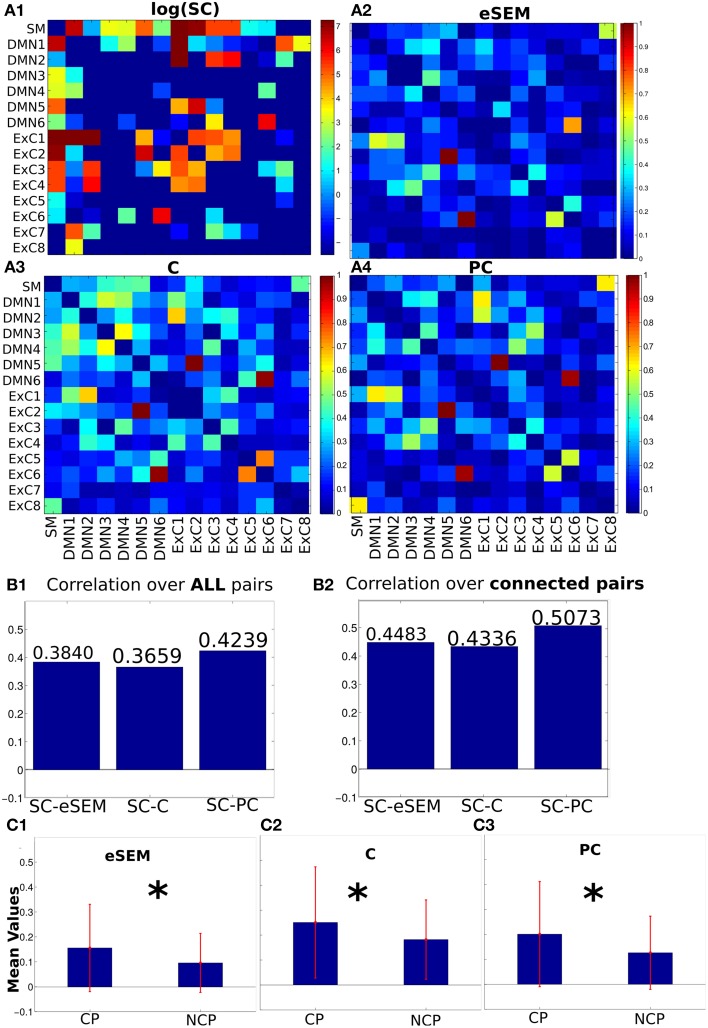
**Structural, effective and functional connectivity matrices (SC, EC and FC, respectively)**. **(A1)** SC matrix calculated by the fiber number. Because many of the values in this matrix are very small, we plotted it in logarithmic scale only to enhance visibility. **(A2–A4)** EC (eSEM) and FC matrices (C and PC), all of them normalized in the [0, 1] range for comparison purposes. **(B)** Correlation-based similarity between SC and eSEM, C and PC, calculated either over all pairs or only on connected pairs. **(C)** Mean values of connectivity matrices separated in two groups: pairs such that they have non-zero fibers between them (structurally connected pairs, CP) and non-connected pairs (NCP). ^*^p<0.01, otherwise means no statistical significance.

Next, the average across subjects SC matrix (Figure 2A1) was computed by averaging the fiber number between pairs of ROIs. Notice that SC is a matrix with many near-zero values. So, it is represented in logarithmic scale just to improve visualization, but all the analyses were performed using the original SC matrix.

Next, three connectivity matrices were calculated for each subject from the rs-fMRI time series: eSEM, C and PC (details in Section 2). Next, an average matrix across subjects was calculated for all matrices. The values of eSEM, C and PC after normalization in the range [0,1] are represented in Figures 2A2–A4. Notice that, unlike C and PC, eSEM provides a non-symmetrical connectivity matrix.

To address the similarity between these matrices, and following previous work (Hagmann et al., 2008; Honey et al., 2009), the Pearson's correlation between the SC entries (vector-wise using all matrix elements) and the corresponding ones for eSEM, C and PC was computed. The three connectivity matrices increased their similarity (based on correlation) with SC on connected pairs, pairs connected with non-zero fibers between ROIs, compared to the situation when all pairs were used for the correlation calculation, i.e., values in Figure 2B2 are bigger than in Figure 2B1. The same results also hold when Spearman's correlations were calculated (Figure S1). It is important to emphasize that these results did not depend on the effect of removing or not the global signal to the time series data. Indeed, similar results than in Figures 2B1,B2 were obtained without global signal removal (Figure S2). Thus, after this simple analysis, we show that the three measures (eSEM, C and PC) are dependent on SC.

We next investigated whether average values of eSEM, C and PC had significant differences between CP and NCP (non-connected pairs). The three connectivity matrices showed bigger (significant) values on CP compared to NCP (Figure 2C), thus indicating that the three methods eSEM, C and PC separated the groups of structurally connected links from those which were not connected. Moreover, PC performed better than eSEM, whilst eSEM and C performed approximately equal.

Next, we addressed the effect that lagged interactions had on eSEM. Thus, when calculating eSEM on lagged-time series, eSEM could not distinguish (i.e., the *p*-value between the two groups was high) between CP and NCP (see **Figure 5**). And this occurred independently on using eSEM or a different model accounting for lagged interactions, here, the method of multivariate GC was used (**Figure 5**). These results indicated that instantaneous measures of interactions (i.e., approaches dealing only with equal-time correlations) are better shaped by SC in comparison to algorithms using temporal information (and this was observed both using eSEM and GC).

**Figure 3:**
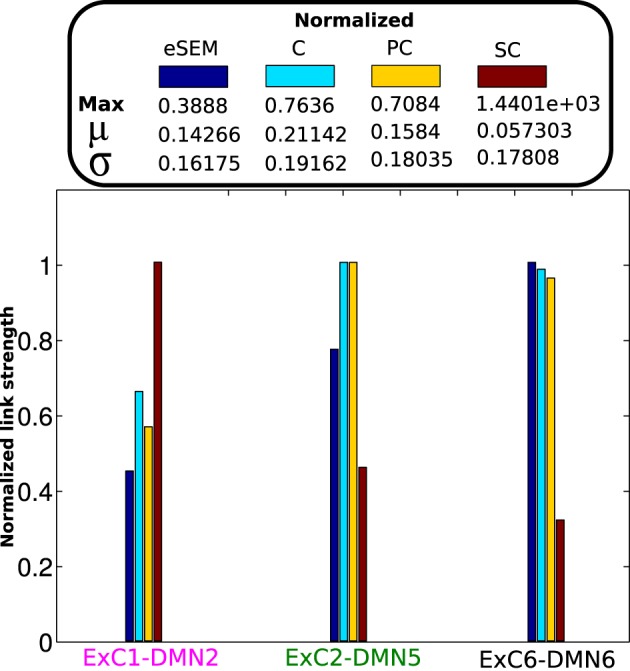
**Connectivity values on specific links**. All matrices eSEM, C, PC, and SC were normalized in the range [0, 1] for visualization purposes. The maximum values used for normalization in each case are shown, as well as the mean (μ) and the standard deviation (σ) values for all matrices.

For a further analysis we looked at the values of SC, eSEM, C and PC on three specific links: the ones with a highest value in each SC, FC, and EC:

- The *structural link*, the pair of ROIs sharing the highest value of SC, which was ExC1-DMN2 (x-label colored in magenta in Figure 3).
- The *functional link*, the pair of ROIs with highest value of C, which was coincident with the pair with maximum PC, that was ExC2-DMN5 (x-label colored in green).
- The *effective link*, the pair of ROIs with highest value of SEM: ExC6-DMN6 (x-label colored in black).

From the structural link, and although the average value of eSEM performed similarly to C (Figure 2B), eSEM gave a significantly smaller value than C and PC, reflecting high relation between ExC1 and DMN2 due to SC. By looking at the functional link, eSEM also provided a high value, indicating that the two areas with neuronal activity most statistical similar each other, ExC2 and DMN5, also had a high directed influence between them. Finally, results on the effective link showed that the link with the highest dynamical influence, from DMN6 to ExC6, also had a high value of C and PC.

**Figure 4:**
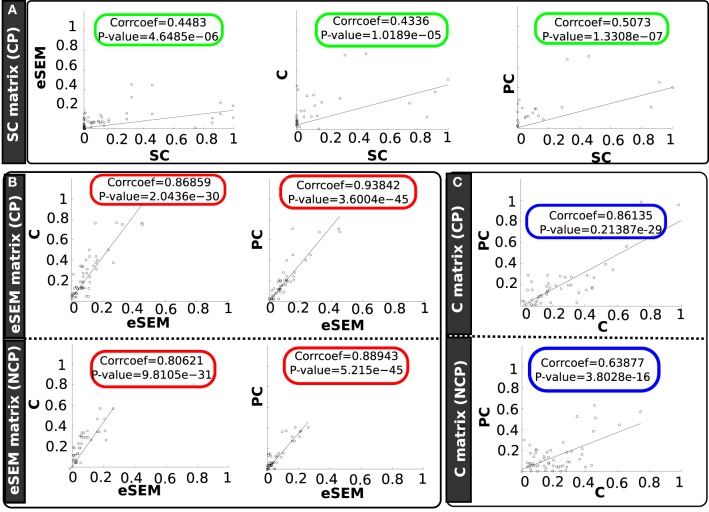
**Scatter plots between different connectivity matrices and separating in two groups: structurally connected pairs (CP) and non-connected pairs (NCP)**. Different panels are showing scatter plots of **(A)** (green rectangles) SC with eSEM, C and PC, **(B)** (red rectangles) eSEM with C and PC, **(C)** (blue rectangles) C with PC.

Beyond results at the level of individual links, scatter plots between the different connectivity matrices (SC, eSEM, C and PC) for all the pairs are shown in Figure 4. The matrices resulting from eSEM, C and PC were significantly correlated with the structural one, SC (rounded green rectangles in Figure 4). Correlation coefficients were 0.44, 0.43, and 0.50 for respectively eSEM, C and PC.

We also found that eSEM matrix was highly correlated with C and PC matrices for both CP and NCP (rounded red circles); indeed, for CP the correlation was equal to 0.86 (for C) and 0.93 (for PC). Thus, on CP pairs, PC and eSEM were approximately equivalent to each other. When looking to NCP, this correlation between eSEM and PC went down to 0.88, still a very high value.

Finally, correlation between C and PC matrices were high for both CP (corr = 0.86) and NCP (corr = 0.63). This is represented by the rounded blue rectangles in Figure 4.

## 4. Discussion

Multiple evidence have shown brain topology (i.e., structure) supporting dynamics (i.e., function) and brain dynamics reinforcing structure via synaptic plasticity (or punishing it via synaptic prunning), but the precise relationship between the two (structure and function) is still challenging (Park and Friston, 2013; Damoiseaux and Greicius, 2015).

A powerful method to approach this problem at the large-scale brain organization is to calculate structural and functional networks and address their mutual relationships (Park and Friston, 2013). Following this strategy, here, we calculated SC, FC, and EC for a very specific brain parcellation, with ROIs covering the entirety of three well-know resting networks, the executive control, the default mode and the sensory motor network. After this brain division, we obtained 15 different ROIs and by performing to the same subject two classes of MRI acquisitions (one structural, one functional) we made a careful comparison between SC (i.e., fiber number connectivity between ROIs), FC (pairwise C and PC connectivities) and EC (by generalizing SEM to its exploratory version eSEM).

We have made use of eSEM for the inference of functional integration; eSEM, although rooted in the SEM framework, is exploratory and can assess influences between brain regions without assuming any implicit model. We have studied how much similar eSEM was to SC, an compared these results with equal-time correlational analysis by calculating both C and PC, which are the leading methods to estimate FC.

In the first part of this study, our results showed that eSEM, in addition to C and PC, were able to significantly separate the set of non-connected pairs in the structural network from the set of connected pairs. Although the PC analysis is slightly the best one in correlating with the strength of structural links, interestingly, for the specific situation of restricting to connected pairs, the eSEM estimation was almost identical to PC (correlation value of 0.93). The fact that eSEM provided a similar correlation with SC to the one achieved by C and PC makes the use of eSEM equally valid as C and PC for FC brain studies.

On the other hand it must be stressed that eSEM also provided information about the case of fiber pairs where information preferably flowed in one direction. These results showed the usefulness of fully connected eSEM inference of directed dependencies between structurally connected ROIs in the human brain.

It is important to emphasize that there are other studies also relating SEM with C and/or PC. Thus, it was shown that PC performed better than SEM in identifying local patterns of interaction detected by SC (Marrelec and Benali, 2009) and that C and PC were suitable candidates to simultaneously analyse SC and FC in the entire brain (Horn et al., 2013); furthermore, this evidence was even stronger when focused on the Default Mode Network, an important RSN with important implications in memory performance. In another study, when SEM was used in combination with DTI data (Voineskos et al., 2012), the authors approached aging and cognitive performance using SC to analyse tract degeneration and SEM to address white matter tract integrity.

**Figure 5:**
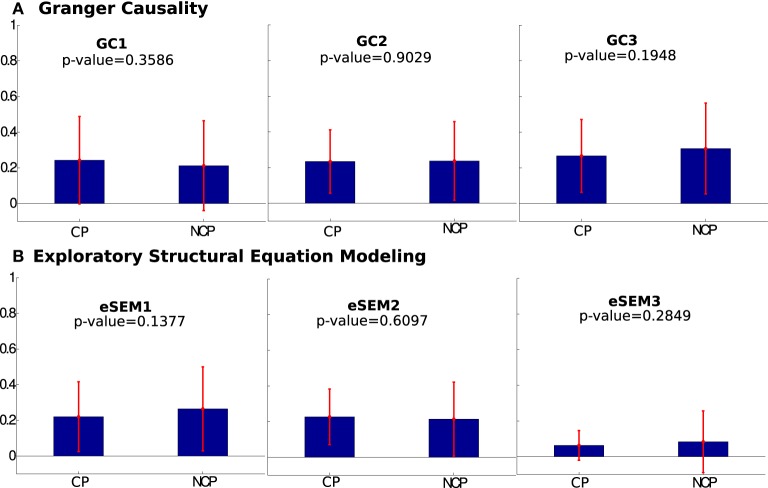
**Mean values of structurally connected pairs (CP) and not connected pairs (NCP) across several lags in (A) Granger Causality and (B) eSEM. eSEM1, eSEM2, and eSEM3 (the same as GC1, GC2, and GC3) refers to lag = {1,2,3} for both eSEM and GC**. Notice that, in all the cases, the differences found between the two groups were not significant according to the *p*-value. So, neither eSEM nor GC distinguished between CP and NCP.

In the second part of our study, we have applied eSEM and multivariate Granger Causality to show that, when lagged time series are considered to estimate EC, the results are much less correlated with SC (Figure 5). This suggests that fast interactions (captured by instantaneous measures of connectivity) are shaped by the structural strength, whilst slower directed functional interactions (those captured by methods relying on temporal correlations) are less shaped by the structural strength. In other words, at slow time scales, the statistical dependencies among ROIs appear to be less related to the details of the underlying structural connectivity.

The fact that the lagged methods found influences between brain regions acting at a time scale equal to the sampling time suggests that the lagged algorithms may be seen as complementary to the standard correlational analysis. The eSEM method, here described, is suitable tool to detect those directed functional interactions which cannot be described merely to the presence of a strong structural connection between brain areas.

*To summarize*, based on the evidence that RSNs are functionally integrated by structural connections (van den Heuvel and Sporns, 2013) here, by building a very simple large-scale brain system consisting of three of those RSNs, and without assuming any implicit connectivity between them, we have shown that eSEM can perform equally well than C and PC in correlating with SC, thus encouraging the use of eSEM for FC studies at rest. Whether this statement still holds during task paradigms needs to be investigated.

## Author Contributions

IE and BM performed MRI acquisitions; IE preprocessed and postprocessed the MRI data; CA performed all simulations; CA, ID, LR, YR, DM, SS, and JC developed the methods; CA, ID, LR, YR, DM, SS, and JC wrote the paper; DM, SS, and JC designed the research; all the authors reviewed the manuscript.

## Funding

Financial support from the Basque Government (BERC 2014-2017) and the Spanish Ministry of Economy and Competitiveness MINECO: BCAM Severo Ochoa accreditation (SEV-2013-0323) to CA and LR; from Ikerbasque: The Basque Foundation for Science and Euskampus at UPV/EHU to JC; from Bizkaia Talent (AYD-000-285) to SS.

### Conflict of interest statement

The authors declare that the research was conducted in the absence of any commercial or financial relationships that could be construed as a potential conflict of interest.

## Data Availability

The SC matrices for each subject and the time series rs-fMRI data are available upon the reader's request.

## Supplementary material

Figure S1 **Pearson's vs. Spearman's correlations**. The same Figures 2B1,B2 are now plotted together with the results from Spearman's correlations for comparison purposes.

Figure S2 **The effect of applying global signal regression vs. not appliying it to the time-series rs-fMRI data**. Similar to Figures 2B1,B2, here we compared the results of applying global signal removal to the time-series data (**A,B** and all other figures in this manuscript), to the results without global signal removal (**C,D**).

## Abbreviations

SC: Structural Connectivity
FC: Functional Connectivity
EC: Effective Connectivity
eSEM: Exploratory Structural Equation Modeling (a non-lagged model)
eSEM1: eSEM with lag = 1
eSEM2: eSEM with lag = 2
eSEM3: eSEM with lag = 3
GC: Granger Causality
GC1: GC with lag = 1
GC2: GC with lag = 2
GC3: GC with lag = 3
C: Correlation
PC: Partial Correlation
RSN: Resting State Network
DMN: Default Mode Network
SM: Sensory Motor
ExC: Executive Control
ROI: Region of Interest
CP: (Structurally) Connected Pairs
NCP: (Structurally) Non-Connected Pairs.

## Appendix

### Effective connectivity (EC) by multivariate granger causality (GC)

Let us first describe bivariate Granger causality (Granger, 1969). Suppose we model the temporal dynamics of a stationary time series {ξ_*n*_}*n* = 1,.,*N*+*m* by an autoregressive model of order *m*:

$$\begin{array}{r} \xi_{n}=\overset{m}{\underset{j=1}{\sum }}A_{j}\;\xi_{n-j}+E_{n}, \end{array}$$

and by a bivariate autoregressive model which takes into account also a simultaneously recorded time series {η_*n*_}*n* = 1,.,*N*+*m*:

$$\begin{array}{r} \xi_{n}=\overset{m}{\underset{j=1}{\sum }}A_{j}^{\prime }\;\xi_{n-j}+\overset{m}{\underset{j=1}{\sum }}B_{j}\;\eta_{n-j}+E_{n}^{\prime }. \end{array}$$

The coefficients of the models are calculated by standard least squares regression; *m* is usually selected according to the Akaike criterion applied to the VAR modeling of the multivariate time series, providing an optimal order of the model (Akaike, 1974).

It can be said that η Granger-causes ξ if the variance of residuals *E*′ is significantly smaller than the variance of residuals *E*, as it happens when coefficients *B*_*j*_ are jointly significantly different from zero. This can be tested by performing an either *F*-test or Levene's test for equality of variances (Geweke, 1982). An index measuring the strength of the causal interaction is $\delta =1-\frac{\left\langle {E^{\prime }}^{2}\right\rangle }{\left\langle E^{2}\right\rangle }$, where ⟨·⟩ means averaging over *n* (note that ⟨*E*⟩ = ⟨*E*′⟩ = 0). Exchanging the roles of the two time series, one may equally test causality in the opposite direction, i.e., to check whether ξ Granger-causes η.

In the conditioned case, let ${\left\{\psi_{n}^{a}\right\}}_{n=1,.,N+m}$, *a* = 1,…,*M*, be *M* other simultaneously recorded time series. When several variables are present in the system, the Granger influence η→ξ must take into account their possible conditioning effect. In this case, it is recommended to treat the data-set as a whole, including the ψ times series in both the autoregressive models for ξ described above. To assess causality in GC, another VAR is learned from data excluding one variable (the candidate driver) from the input set of variables. Then, an *F*-test is applied to assess significance of the variance reduction due to the inclusion of the candidate driver variable. The conditioned Granger causality η → ξ measures the reduction in the variance of residuals going from one to other of the following two conditions: (i) all variables ψ are included in the model and (ii) all variables ψ and the variable η are included. Conditioning on the remaining variables allows to discard indirect interactions that would be recognized as direct by the pairwise approach. We refer the reader to Stramaglia et al. (2014) for a discussion on advantages and pitfalls of pairwise and conditioned Granger causality. In this paper we will refer to GC1, GC2, GC3 when discussing the application of GC with *m* = 1, 2, 3 respectively.
