# Supplementary figures and images for: Lagged and instantaneous dynamical influences related to brain structural connectivity
Source: Front Psychol. 2015 Jul 21;6:1024. doi: 10.3389/fpsyg.2015.01024 (PMC4508482; doi:10.3389/fpsyg.2015.01024)

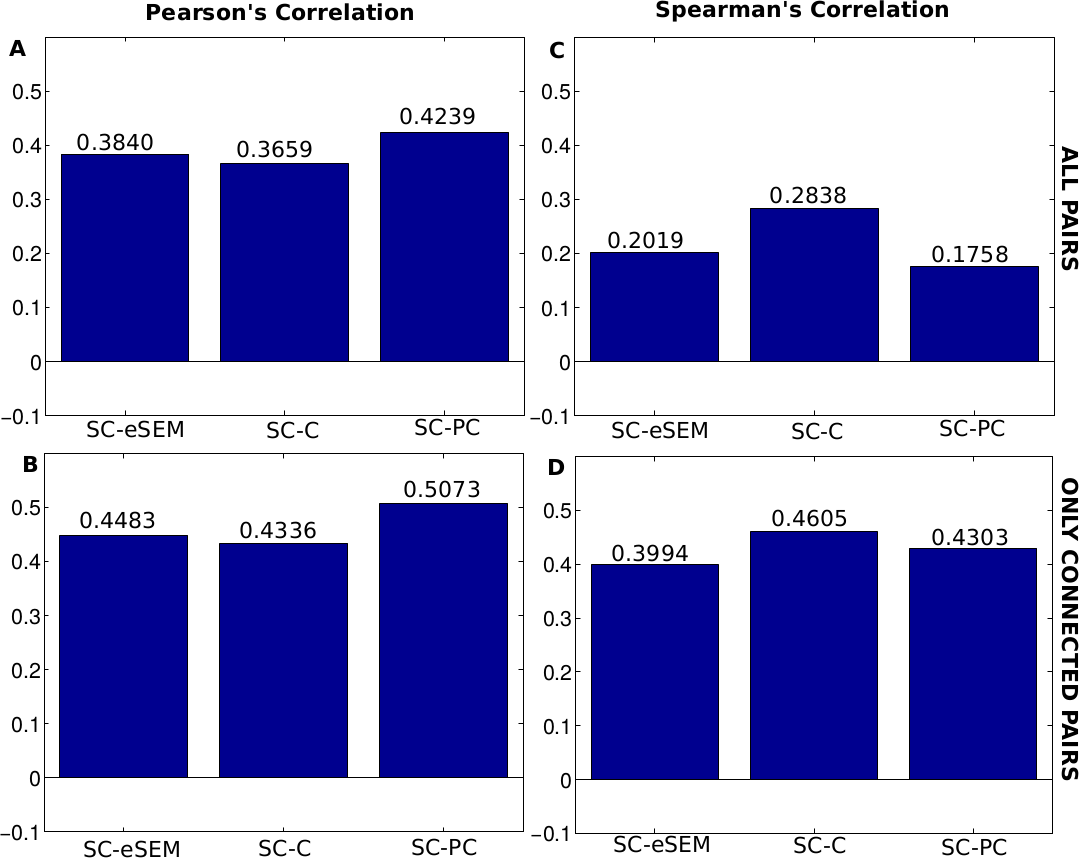

Supplement: Figure S1 — Pearson's vs. Spearman's correlations. The same Figures 2B1,B2 are now plotted together with the results from Spearman's correlations for comparison purposes. [file Image1.TIFF]

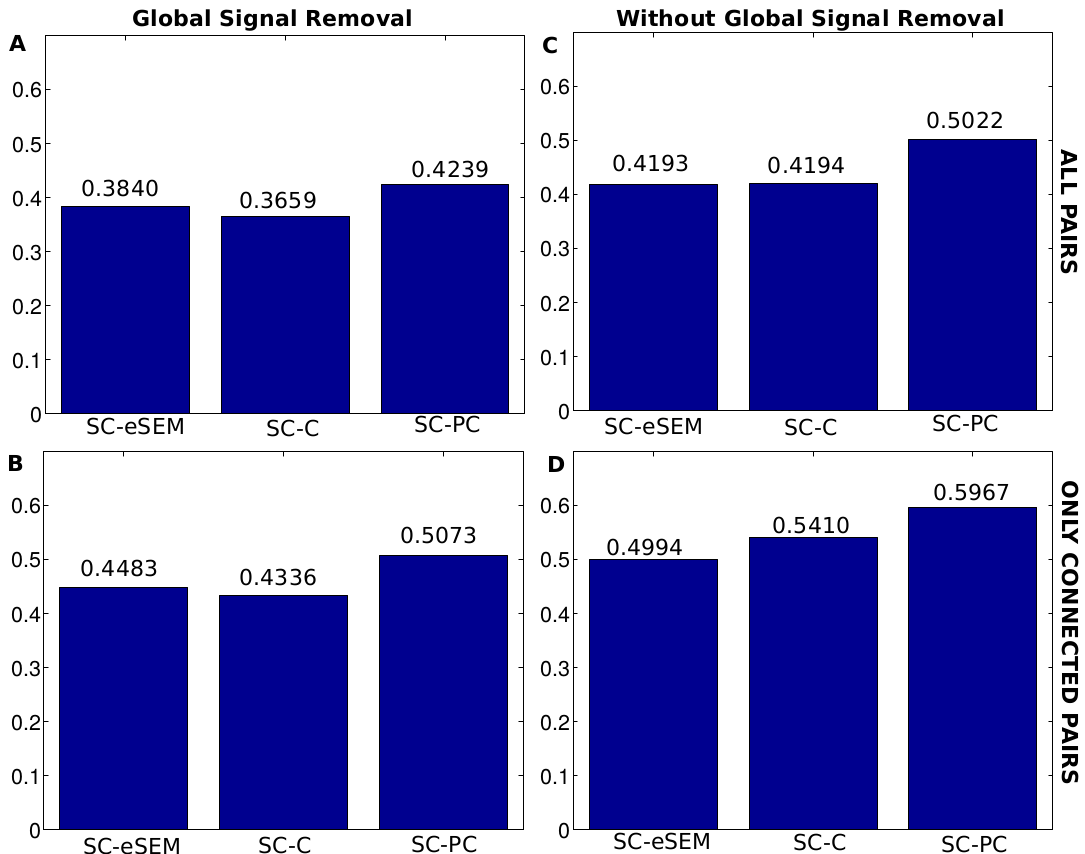

Supplement: Figure S2 — The effect of applying global signal regression vs. not appliying it to the time-series rs-fMRI data. Similar to Figures 2B1,B2, here we compared the results of applying global signal removal to the time-series data (A,B and all other figures in this manuscript), to the results without global signal removal (C,D). [file Image2.TIFF]
